# So-Called Ozomulsion

**Published:** 1897-07

**Authors:** 


### SO-CALLED OZOMULSION.

The study of modern advertising methods is of considerable interest. The methods which are pursued by those who present therapeutic agents to the profession are naturally different from the ones adopted by advertisers to the laity at large. It is rare that we have an oportunity of seeing products advertised by reputable medical journals, and at the same time pushed among the people in such ways as are used by T. A. Slocum, M. C., whatever these letters may mean. The following printed circular letter, with the address written in ink, has been sent to several persons in and about Cleveland who have bought the so-called "Ozomulsion," and is evidently a stock communication:

MEDICAL COUNCIL AND LABORATORY DEPARTMENT.
T. A. SLOCUM, M. C.

Office Hours: 10 A. M. to 4 P. M.

NO. 98 PINE STREET,

NEW YORK, June 4, '97.

—— ———, Esq.,

DEAR SIR: It is now about a month since I had the pleasure of sending you samples of my Remedies in response to your application; and I sincerely hope that they have already done you good.

Nearly all the people to whom I sent the remedies at the same time I sent yours, have written to say they are improving, and have ordered a further supply.

I am exceedingly pleased to hear such good news although it was no better than (knowing the nature of my medicine) I had reason to expect. I look forward to hearing in a few months more, that most, if not all of them are entirely well—rid forever of the dreadful disease which was hurrying them to the grave. I have written to each and every one of them a letter of congratulation and advice. Not having received any word from you to this date, I began to ask myself the reason why. Perhaps you did not have consumption and are already relieved of the ailment you suffered from. Certain rare forms of dyspepsia are very close counterfeits of consumption and deceive many. They are always speedily and radically cured by my Psychine. Yours may be a case of this kind. If so, it was for you a fortunate mistake, and I am glad of it for your sake. But the chances are a hundred to one against that theory.

Consumption is the most deceitful, as well as the most fatal, of all diseases. To trust to its mercy is the wildest of folly. Before you begin to realize your danger, it grasps you with a power no human means can shake.

To delay and to hesitate even when you *only suspect* the presence of consumption is like trusting a hungry tiger.

The churchyards are full of people who said of this white plague, I didn't think it was anything serious. Any physician will assure you of the truth of these solemn words of warning.

I now offer you a cure which if employed faithfully and as directed will prove a genuine and lasting one.

My system of medicine is the result of the study and experience of my whole life. It has stood the strictest examination by physicians and druggists, and what is more to the purpose, it has cured countless cases of true consumption.

I pledge you my word as a man who has never trifled with serious things, that the quantity named in the enclosed proposition will effect a permanent cure if my instructions are observed.

Faithfully,

T. A. SLOCUM, M. C.

P. S.—I enclose a notice from the *United States Journal of Medicine*, which may give you confidence in my ability and Remedies to cure you.

Enclosed with this ingenuous appeal is the following special cure offer:

NO. 90273. 98 Pine St., NEW YORK.

MEDICAL LABORATORY OF T. A. SLOCUM, M. C.

SPECIAL CURE OFFER.

*HAVING RECEIVED TEN DOLLARS (10.00), we hereby agree to send to.................... Cleveland, Ohio., three large bottles of Psychine, three large bottles of Ozomulsion, one large bottle of Coltsfoote Expectorant, and four boxes of Lazy Liver Pills, which supply will effect a permanent cure of the Malady from which the invalid is now suffering, and if the medicine fails to effect a cure or give satisfactory benefit, when taken according to directions, this Company, at its option, will return the money or send more medicine FREE.*

*New York..................189.* .......................

It is hardly necessary to comment on the above literature. It will suffice to say that Ozomulsion is advertised in many of the medical journals of the country and has been supplied in sample bottles to physicians. Aside from the fact that The Ozomulsion Company attempts to supplant the physicians in cases in which Ozomulsion is used instead of cod-liver oil, the dishonesty of such direct promises as are made by them must be apparent. It is the duty of the medical press of this country to give the widest publicity to the methods of this company, and to refuse to carry the advertisements of such a firm.—*Cleveland Journal of Medicine.*

